# Sintra Grows Healthy: development and implementation of a food literacy curriculum for primary schools

**DOI:** 10.1017/S1368980022000180

**Published:** 2022-05

**Authors:** Telma Nogueira, Raquel J Ferreira, Marta Sócrates, Vitória Dias da Silva, Mariana Liñan Pinto, Rute Borrego, Joana Sousa

**Affiliations:** 1 Laboratório de Nutrição, Faculdade de Medicina, Universidade de Lisboa, Avenida Professor Egas Moniz, Edifício Egas Moniz, Ala C, Piso 2, Lisboa 1649-028, Portugal; 2 Instituto de Saúde Ambiental, Faculdade de Medicina, Universidade de Lisboa, Avenida Professor Egas Moniz, Edifício Egas Moniz, Lisboa 1649-028, Portugal; 3 Câmara Municipal de Sintra, Departamento de Educação, Juventude e Desporto, Largo Dr. Virgílio Horta, Sintra, Portugal; 4 Escola Superior de Tecnologia da Saúde de Lisboa, Instituto Politécnico de Lisboa, Avenida D. João II, Lisboa, Portugal

**Keywords:** Curriculum, Food literacy, Nutrition education, Health promotion, School

## Abstract

**Objective:**

Describe the process of development and implementation of *Health at the Table* – a food literacy curriculum for primary school aged children.

**Design:**

Through a community-based research process, *Health at the Table* development and implementation took place in four stages: exploratory study, production, implementation and monitoring.

**Setting:**

Primary schools of Sintra’s municipality, Portugal.

**Participants:**

Children (6–10 years), teachers, school staff and children’s legal guardians of three primary schools during the pilot project and eight primary schools in the second year.

**Results:**

During the needs assessment phase, 99·1 % (*n* 341) of the children’s legal guardians, 100 % (*n* 34) of the teachers and 100 % (*n* 19) of the school staff considered that the school plays an important or very important role in children’s food literacy (stage 1). During the pilot project, a manual with sixty session plans was developed (stage 2). In the second year, *Health at the Table* was implemented by seventy-two trained teachers during one school year (stage 3). Most of the teachers agreed that the curriculum was appropriate (69·2 %) and that children developed health, wellness/well-being and environmental skills (83·1 %). Most of the children said they had learned about healthy eating (86·3 %) and claimed to eat healthier since the *Health at the Table* implementation (58·9 %) (stage 4).

**Conclusions:**

*Health at the Table* is a food literacy curriculum that can be reproduced in similar contexts in a sustainable way. The need to combine educational strategies with a healthy school food environment is reinforced to increase effectiveness in tackling childhood obesity.

Obesity is a complex system of distinct and interrelated factors associated with significant short and long-term negative consequences for individuals and societies^(1–3)^. Thus, preventing obesity is a public health priority^(4)^.

In Portugal, the prevalence of children under 10 years old with obesity and pre-obesity is, respectively, 7·7 % and 17·3 %^(5)^; 12 % of children aged 6–8 years are obese and 29·6 % are overweight^(6)^. In the primary schools of Sintra’s municipality, the prevalence changes to 12·6 % obese and 23 % overweight^(7)^, a high percentage when compared with other regions in Europe^(7)^.

International^(4,8,9)^ and national^(10,11)^ guidelines state that municipalities are promising spaces for improving children’s nutritional status and that they play a crucial role in tackling childhood obesity.

Schools constitute an important setting for promoting health, food literacy and nutrition education^(12)^, establishing healthy behaviours^(13)^, protecting and supporting good nutrition in children and their families and communities^(14,15)^. Food literacy and nutrition education in schools can be incorporated in different ways from the basic curriculum to extracurricular activities^(12,14,16)^. In addition, school is an excellent setting for communicating health-promoting messages to the entire school community^(14)^. A health-promoting school implements structured and systematic plans considering well-being, health, and social development for students, teaching and non-teaching staff^(17)^, principles supported by the Convention on the Rights of the Child^(18)^, the Ottawa Charter^(19)^ and the Schools for Health in Europe Network Foundation^(20)^.

Health-promoting interventions are more effective if they are initiated early^(21)^ and if there is agreement on the attitudes and responsibility of the family, school and community^(14,16)^. Also, there is some evidence of the effectiveness of school interventions to improve weight status and increase physical activity, when supported by the health-promoting school model^(22)^.

The National Health Education Referential^(23)^ is a flexible tool of voluntary implementation that establishes topics and objectives for health education in the school environment, namely in the context of nutrition education according to schooling year.

There are several nutrition interventions described in the literature, but the authors found no detailed manuscripts regarding the development and implementation of specific food literacy tools. To strengthen food literacy and nutrition education in primary schools, the Municipality of Sintra implemented an intervention to promote healthy lifestyles – Sintra Grows Healthy^(24)^. Therefore, this study aims to describe the process of development and implementation of *Health at the Table* – a food literacy curriculum for primary school-aged children, specifically developed in the context of the larger intervention ‘Sintra Grows Healthy’^(24)^.

## Methods

The study was developed using data from the Sintra Grows Healthy intervention^(24)^. Sintra Grows Healthy intervention follows a community-based participatory research methodology and focuses on school community actors. A food and nutrition curriculum named *Health at the Table* is one of the axis of the intervention and consists of weekly sessions of food literacy and nutrition education^(24)^. For the implementation of this axis, the Sintra Grows Healthy team developed a food literacy curriculum and a specific manual to support its implementation, whose process is hereby described.

### Participants

During the pilot project, 467 children and legal guardians, 27 teachers and 19 school staff coming from 22 classes of three primary schools belonging to one school cluster participated in *Health at the Table*. During the second school year of intervention, the number of participants increased to 1734 children and legal guardians, 98 teachers and 40 school staff coming from 77 classes of eight primary schools belonging to three school clusters.

### Procedure

**Fig. 1:**
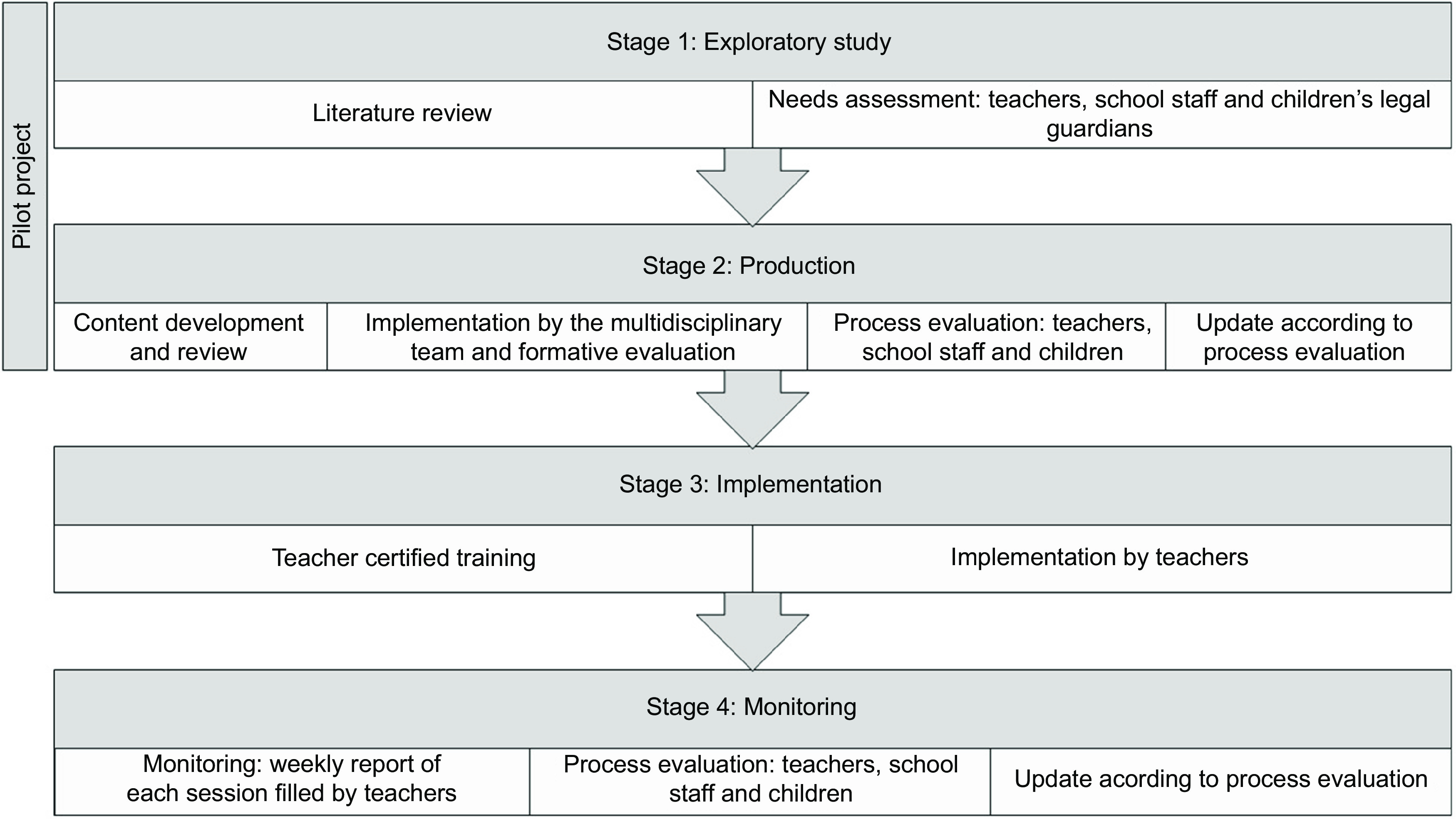
Health at the Table development and implementation Flowchart

The development and implementation of *Health at the Table* began in the 2017–2018 school year and took place in four stages (Fig. 1).

### Stage 1 – exploratory study

The first stage entailed the completion of a needs assessment questionnaire, consisting of both closed and open-ended questions, in one school cluster with three primary schools and twenty-two classes. The questionnaire was administered to children’s legal guardians (20 items), teachers (23 items) and school staff (21 items). The close-ended questions intended to evaluate healthy eating habits and attitudes in primary school children, the importance of food literacy as well as the school setting and its significant role for health promotion (e.g. ‘School plays a key role in education and promoting healthy eating behaviours.’), using a 5-point Likert scale. The open-ended questions aimed to collect suggestions and improvement opportunities.

Simultaneously, a literature review on optimal food and nutrition education practices for primary school children was conducted to gather evidence to support the curriculum development^(20,22,23)^.

### Stage 2 – production

Six thematic areas and their respective objectives were defined aligned with the National Health Education Referential^(23)^: (i) Food and culture; (ii) Food, nutrition and health; (iii) Food and emotions; (iv) Food cycle: from the producer to the consumer; (v) Safe cooking and (vi) Food sustainability.

A multidisciplinary team, with registered dietitian/nutritionists, psychologists and primary school teachers, developed, applied and reviewed session plans for each thematic area. These session plans included both theoretical and practical activities, such as recognising healthy or unhealthy snacks, consulting nutrition labelling, checking the adequacy to children’s energy and nutritional needs or presenting solutions to reduce food waste at school. The manual provides the contents organised for each schooling level allowing connection with the basic curriculum subjects (Maths, Science and Portuguese).

*Health at the Table* was implemented by a multidisciplinary team as an extracurricular activity.

At the end of the school year, the process evaluation was performed. Children answered a self-reported three-item questionnaire, using a three-choice smile scale: if they enjoyed *Health at the Table*, if they learnt something about healthy eating and if their eating habits became healthier.

In the same way, similar questionnaires were applied, both in close and open-ended questions, to children’s legal guardians (6-item), teachers (12-item) and school staff (6-item). The close-ended questions, on a 5-point Likert scale, included specific questions related to *Health at the Table* (e.g. ‘Children enjoyed *Health at the Table*?’). The open-ended questions aimed to collect suggestions and improvement opportunities.

### Stage 3 – implementation

In the second school year, after the pilot project, *Health at the Table* was no longer implemented as an extracurricular activity but integrated into the school curriculum as ‘complementary offer’ — a mandatory curriculum component established in Portuguese law. Complementary offer is considered a new mandatory attendance subject (not covered in the basic curricular matrices) that has its own identity and curriculum documents^(25)^. Through curricular flexibility and articulation, the complementary offer allows the curriculum to be enriched with knowledge, skills and attitudes that contribute to achieving the competencies provided in the Profile of Students after Leaving Compulsory Education, particularly in the area of wellness, health and environment^(26)^. Thus, in the second school year, *Health at the Table* was put into practice by the responsible teacher of each class. To ensure that the content was properly delivered to children, Sintra Grows Healthy developed a 50-h certified training for all teachers. The training was divided into six modules, one for each thematic area in the curriculum, and was conducted by trainers certified by the National Scientific-Pedagogical Council of Continuing Education^(27)^. *Health at the Table* was applied by the trained teachers of three school clusters.

### Stage 4 – monitoring

The monitoring occurred through a weekly submission form into an online platform for each food literacy session applied by the teacher. In this submission, teachers answered seventeen questions (e.g. date of application, children’s competencies achieved, activity adequacy and improvement opportunities). In addition, a similar process evaluation was performed, as described in stage 2. With the information obtained in stage 4, the curriculum is continuously reviewed and updated by the Sintra Grows Healthy team. Also, this monitoring will allow to perform the impact assessment considering other facets of process evaluation such as responsiveness, dose, reach, fidelity and quality.

## Results

A needs assessment (Stage 1) was conducted in twenty-two classes of three primary schools belonging to one school cluster. Questionnaires were applied to all teachers (*n* 34) and school staff (*n* 19), and children’s legal guardians (*n* 344). The response rate was 100 % for teachers and school staff, and 73·7 % for children’s legal guardians. All teachers (100 %) and 94·7 % of school staff agreed that children in primary schools are in a crucial period to develop food literacy and 97·1 % of teachers and all school staff (100 %) agreed that their school could contribute significantly to nutritional education and to promote healthy eating behaviours. Furthermore, 99·1 % of the children’s legal guardians, 100 % of the teachers and 100 % of the school staff considered that the school plays an important or very important role in children’s food literacy. Also, 97·6 % of the children’s legal guardians, 100 % of the teachers and 100 % of the school staff considered that the school plays an important or very important role in preventing childhood obesity. In addition, all teachers considered that there is an opportunity to work on healthy eating topics at school. Related to the thematic areas, most children’s legal guardians considered important or very important to work on these topics at school: Food and culture (98·0 %), Food, nutrition, and health (99·1 %), Food cycle: from the producer to the consumer (95·3 %), Safe cooking (98·5 %) and Food sustainability (94·7 %).

**Graph 1:**
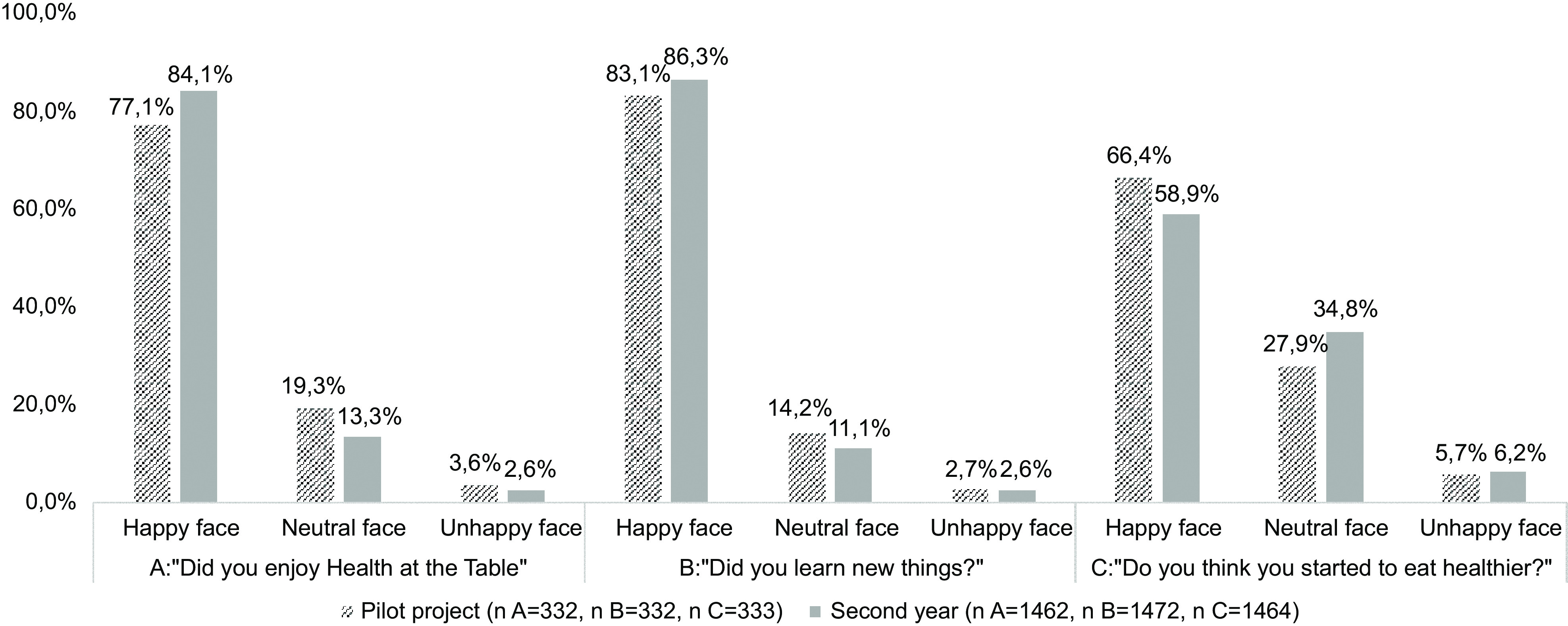
Results of process evaluation applied to children

**Graph 2:**
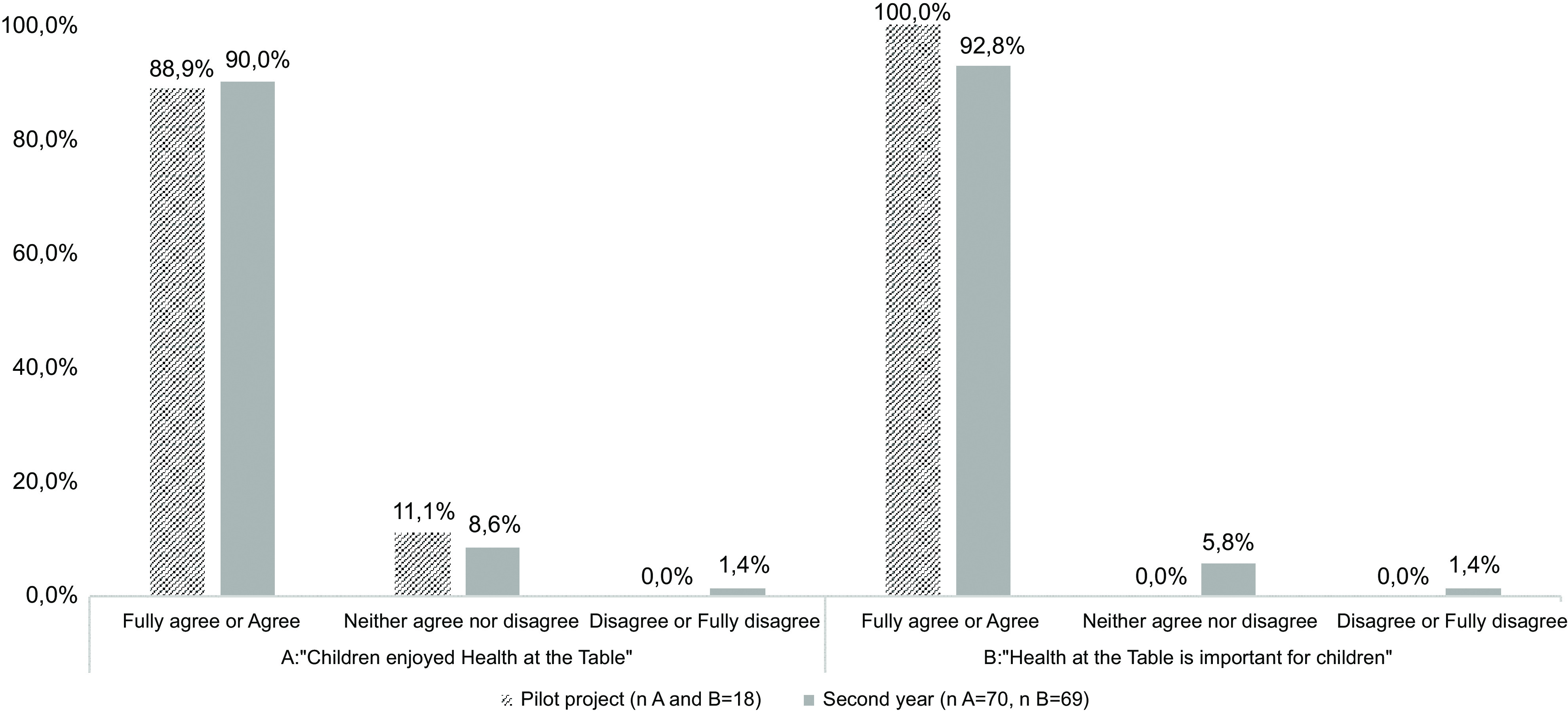
Results of process evaluation applied to teachers

**Graph 3:**
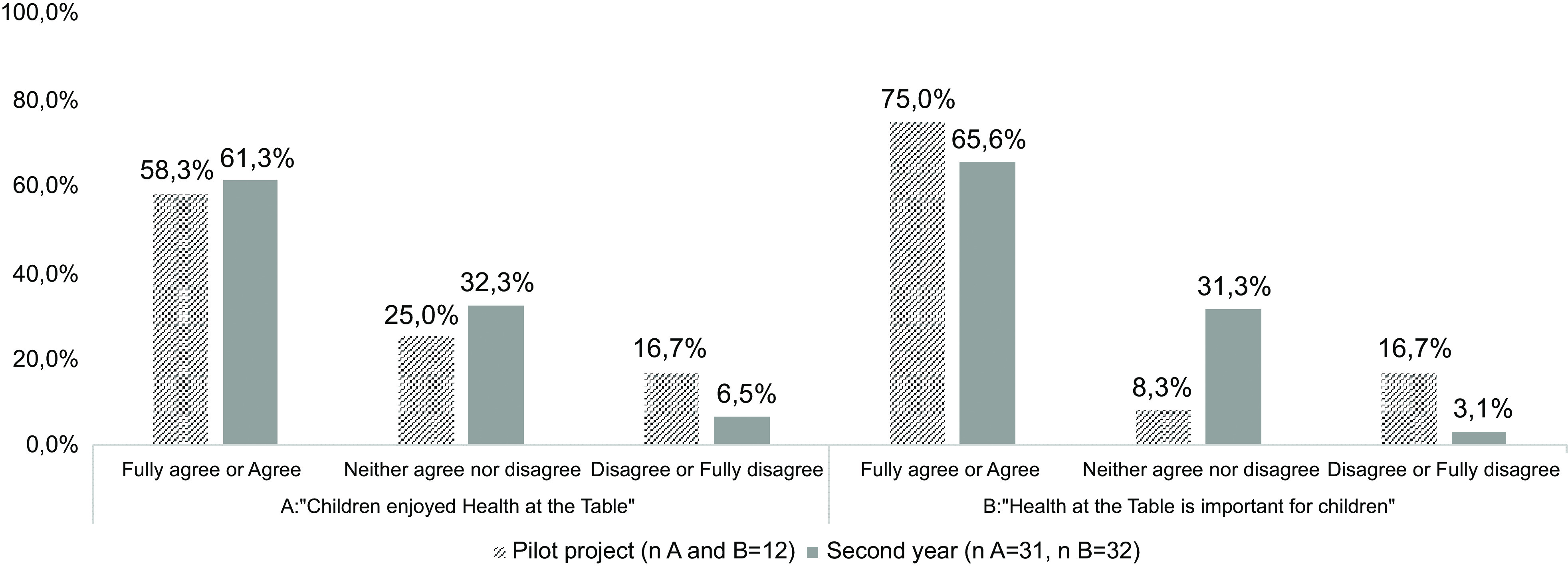
Results of process evaluation applied to school staff

**Graph 4:**
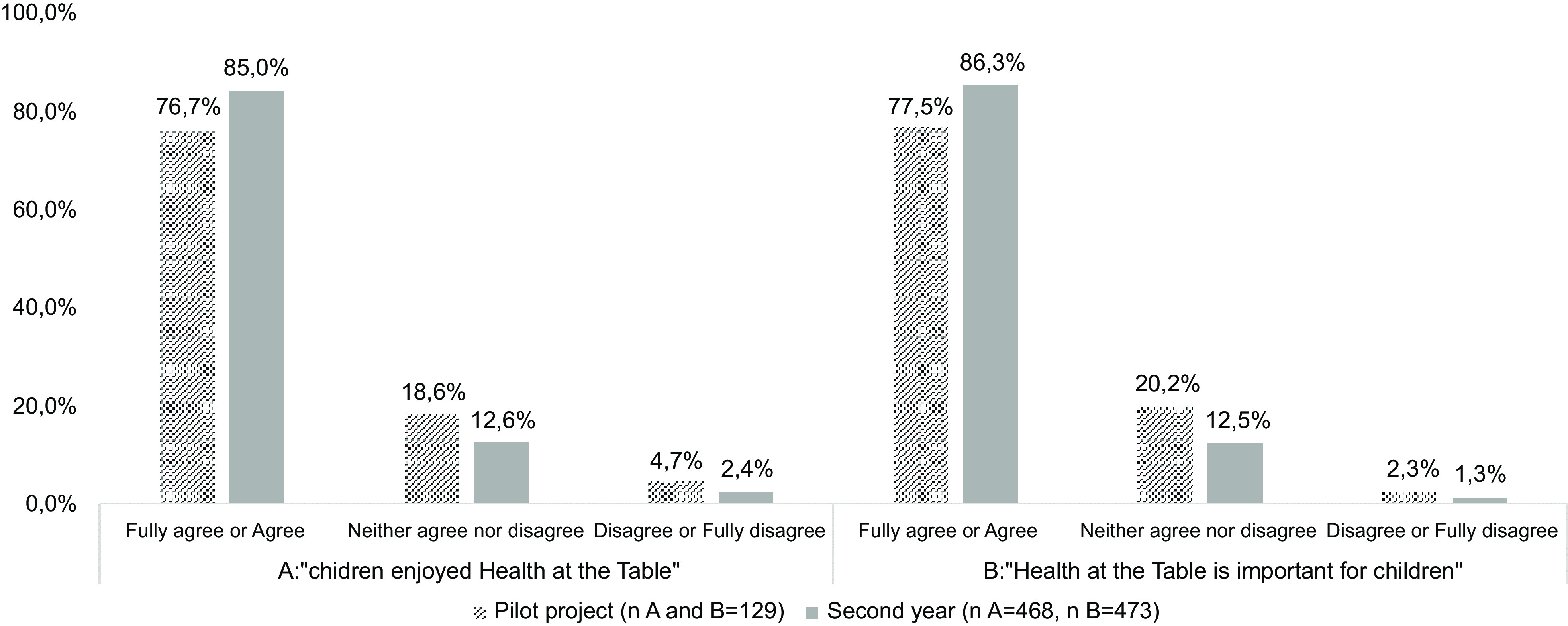
Results of process evaluation applied to children’s legal guardians

During the pilot project, a sixty-session plan manual was developed (Stage 2), divided by six thematic areas, and implemented in twenty-two classes of one school cluster. The results of specific questions of the process evaluation completed in the pilot project are presented in Graphs 1–4. In addition, in the pilot project, 88·9 % (*n* 16) of the teachers agreed that children developed health, wellness and environmental skills with *Health at the Table*. Moreover, 66·7 % (*n* 10) of the teachers perceived improvements in children’s eating behaviours at the school canteen. Most children’s legal guardians (61·2 %, *n* 79) fully agreed or agreed that *Health at the Table* is important for them as educators.

In the second year, when *Health at the Table* became ‘complementary offer’ (Stage 3), seventy-two teachers received a 50-h certified training to apply the food literacy curriculum in three school clusters.

The results of specific questions of the process evaluation completed in the second year (Stage 4) are also presented in Graphs 1, 2, 3 and 4. Regarding teachers, 83·1 % (*n* 54) stated that children developed health, wellness and environmental skills with *Health at the Table*, and 69·2 % (*n* 36) agreed the curriculum was adequate. The percentage of children’s legal guardians that fully agreed or agreed that *Health at the Table* is important for them as educators increased in the second year (76·5 %, *n* 361).

## Discussion

The theoretical–practical methodology of the *Health at the Table* curriculum, including activities that stimulate observation, discussion, actions and practice in real-life settings, might have contributed to the improvement of children’s food literacy and food and nutrition education^(28)^. Evidence shows that better results can be obtained from a learning through playing approach^(29)^, and cooking education may positively influence children’s food-related preferences, attitudes and behaviours^(30)^.

The involvement of the school community in the participatory development of the curriculum, whenever considering their needs, motivates its application^(31)^.

As outlined in the literature, the school community agrees that children in this age group are in a critical period to receive food literacy and nutrition education, and school context is an opportunity to work on issues and attitudes associated with healthy eating^(21,32,33)^.

It was perceived by the school community that most children enjoyed and developed skills with *Health at the Table*. The application of *Health at the Table* by teachers ensures curricular flexibility and articulation, allowing the curriculum to be enriched with the knowledge, skills and attitudes as foreseen by national^(23,26)^ and international guidelines^(20,34)^.

Most children (66·4 % in the pilot project and 58·9 % in the following school year) perceived having healthier eating habits after the intervention. There is evidence that food literacy favours the improvement of children’s eating habits when transmitted in the school environment and allied to the involvement of the whole school community and local governments^(35,36)^.

A higher percentage of children (83·1 % in the pilot project and 86·3 % in the following school year) reported having learnt new things related to food and nutrition after the intervention. The difference between the percentage of children that perceived having healthier eating habits and that reported having learnt new things might reinforce the argument that knowledge is a necessary but not sufficient factor for changes in behaviours^(37)^.

Evidence on interventions in this field suggests that multi-component approaches^(17,32,38–40)^, that integrate the whole community^(41)^, long-term^(42,43)^ and supported by sustainable models^(44)^ are more likely to succeed^(45)^. Sintra Grows Healthy, that includes *Health at the Table*, presents itself as an intervention that fulfils these criteria.

The need for prior training of teachers to implement this curriculum could be considered as a limitation. However, it is crucial to ensure the transmission of accurate content according to scientific evidence. Also, the study was conducted in eight primary schools in Sintra, limiting the generalisability.

*Health at the Table* presents itself as an innovative curriculum, through its participatory development methodology. In addition, its success lies in its constant monitoring, which allows short cycles of evaluation–planning–intervention, contributing to meet the emerging needs and interests of the school community.

## Conclusion

*Health at the Table* is a food literacy curriculum, that could be sustainably reproducible in similar contexts. Comprehensive school-based nutrition interventions involve the school community and address multiple components. Thus, the implementation of Sintra Grows Healthy includes the integration of food literacy in the curriculum **–** *Health at the Table –* as well as the development of policies capable of modifying the school food environment, to support and facilitate the adoption of healthy behaviours, characteristic of a health promoting school.
